# Evaluation of the sensitivity of two 3D diode array dosimetry systems to setup error for quality assurance (QA) of volumetric‐modulated arc therapy (VMAT)

**DOI:** 10.1120/jacmp.v14i5.3828

**Published:** 2013-09-06

**Authors:** Guangjun Li, Sen Bai, Nianyong Chen, Lansdale Henderson, Kui Wu, Jianghong Xiao, Yingjie Zhang, Qingfeng Jiang, Xiaoqin Jiang

**Affiliations:** ^1^ Department of Radiation Oncology, Cancer Center West China Hospital Sichuan University Chengdu Sichuan China; ^2^ Center for Radiation Physics and Technology, Cancer Center West China Hospital Sichuan University Chengdu Sichuan China; ^3^ Department of Neuroscience University of Virginia Charlottesville VA USA

**Keywords:** VMAT, setup error, patient‐specific QA, 3D diode array

## Abstract

The purpose of this study is to evaluate the sensitivities of 3D diode arrays to setup error for patient‐specific quality assurance (QA) of volumetric‐modulated arc therapy (VMAT). Translational setup errors of ±1,±2, and ±3 mm in the RL, SI, and AP directions and rotational setup errors of ±1° and ±2° in the pitch, roll, and yaw directions were set up in two phantom systems, ArcCHECK and ${\text{Delta}}^{4}$, with VMAT plans for 11 patients. Cone‐beam computed tomography (CBCT) followed by automatic correction using a HexaPOD 6D treatment couch ensured the position accuracy. Dose distributions of the two phantoms were compared in order to evaluate the agreement between calculated and measured values by using γ analysis with 3%/3 mm, 3%/2 mm, and 2%/2 mm criteria. To determine the impact on setup error for VMAT QA, we evaluated the sensitivity of results acquired by both 3D diode array systems to setup errors in translation and rotation. For the VMAT QA of all patients, the pass rate with the 3%/3 mm criteria exceeded 95% using either phantom. For setup errors of 3 mm and 2°, respectively, the pass rates with the 3%/3 mm criteria decreased by a maximum of 14.0% and 23.5% using ArcCHECK, and 14.4% and 5.0% using ${\text{Delta}}^{4}$. Both systems are sensitive to setup error, and do not have mechanisms to account for setup errors in the software. The sensitivity of both VMAT QA systems was strongly dependent on the patient‐specific plan. The sensitivity of ArcCHECK to the rotational error was higher than that of ${\text{Delta}}^{4}$. In order to achieve less than 3% mean pass rate reduction of VMAT plan QA with the 3%/3 mm criteria, a setup accuracy of 2 mm/1° and 2 mm/2° is required for ArcCheck and ${\text{Delta}}^{4}$ devices, respectively. The cumulative effect of the combined 2 mm translational and 1° rotational errors caused 3.8% and 2.4% mean pass rates reduction with 3%/3 mm criteria, respectively, for ArcCHECK and ${\text{Delta}}^{4}$ systems. For QA of VMAT plans for nasopharyngeal cancer (NPC) using the ArcCHECK system, the setup should be more accurate.

PACS numbers: 87.55.ne, 87.55.Qr, 87.55.km

## I. INTRODUCTION

Volumetric‐modulated arc therapy (VMAT) is a new intensity‐modulated radiotherapy (IMRT) technology with single or multiple gantry arcs that achieves appropriate dose‐target conformity and permits critical organ sparing. VMAT delivers radiation via dynamic multileaf collimator (MLC) motion, and allows for variable dose rates, gantry speed modulation, and collimator rotation.^(1)^ Thus far, VMAT has been used to treat various tumor sites, including head and neck,^2^, ^3^, ^4^, ^5^ lung,^5^, ^6^, ^7^, ^8^ prostate,^(^ ^4^, ^5^ ^,^ ^9^, ^10^, ^11^, ^12^ ^)^ rectum,^(^ ^5^ ^,^ ^13^ ^)^ cervix uteri,^(14)^ spinal metastases,^(^ ^15^ ^,^ ^16^ ^)^ and brain metastases.^(17)^ However, the dose calculation and the implementation of VMAT plans are highly complex. It is, therefore, essential to perform patient‐specific quality assurance (QA) of VMAT plans.^(18)^

Various types of dosimetry systems exist for dose verification, including gel dosimetry,^(^ ^19^ ^,^ ^20^ ^)^ water phantoms with film and ion chambers,^(21)^ online 2D detector arrays,^22^, ^23^, ^24^, ^25^ Monte Carlo‐based frameworks,^(26)^ and 3D diode arrays.^27^, ^28^, ^29^ With the exception of the 3D diode arrays, these QA systems are limited either by single‐plane measurements of the dose distribution or increased off‐line processing time for measured data.^(27)^ Two commercial 3D diode array systems, ArcCHECK (Sun Nuclear, Melbourne, FL) and ${\text{Delta}}^{4}$ (ScandiDos AB, Uppsala, Sweden), were applied for dose verification in IMRT and VMAT.

In our clinical practice, we used ArcCHECK and ${\text{Delta}}^{4}$ for QA of VMAT plans. During QA measurements, the phantoms were positioned such that the crosslines on the surface aligned with the room lasers. In practice, we noted that the registration errors (e.g., 2 mm) of the room lasers and the radiation isocenter of the linacs affected the phantom positioning error, and thereby obviously influenced the dose verification of VMAT. However, unlike most other commercial systems, the analysis software available in the ArcCHECK and ${\text{Delta}}^{4}$ systems is unable to correct for positioning errors. Therefore, it is crucial to determine the sensitivity of the 3D detector arrays to setup error for QA of VMAT plans. In one case, Letourneau et al.^(27)^ assessed the sensitivity of the prototype of the ArcCHECK dosimetry system to phantom translational setup error in the right‐left and anterior‐posterior directions and the results demonstrated that the diode array sensitivity to setup error is strongly dependent on the patient‐specific VMAT plans. However, the effect of the rotational setup error on ArcCHECK and other 3D detector arrays with various detector positions is not fully understood. In this study, we examined the sensitivities of ArcCHECK and ${\text{Delta}}^{4}$ to translational and rotational setup errors in all directions for patient‐specific QA of VMAT plans.

## II. MATERIALS AND METHODS

### A. Patients' plan selection

Eleven patients requiring VMAT plans of differing complexity for cancers, including esophageal, prostate, cervix uteri, rectal, and nasopharyngeal cancer (NPC), were selected for this study. The VMAT plans were designed using a commercial 3D treatment planning system (Pinnacle v9.0, Philips Medical, Madison, WI) with a SmartArc optimization algorithm.^(30)^ Patients' characteristics and planning states are summarized in Table 1. The plans for NPC had two full arcs with one control point per 4°, and the plans of other cancer sites had only one full arc with one control point per 4°. All TPS calculations in this study were done with a dose grid resolution of 2 mm.

**Table 1:** Patient characteristics and planning states. Using the SIB technique, two or three dose levels are defined for each patient, except for patients with rectal cancer

*Disease Site*	*Number of Patients*	*Dose Levels*	*Monitor Units*	*Average Field Width (mm)*	*Average Leaf Travel* ^(a)^ (mm)	*Average Leaf Travel Speed* ^(b)^ (mm/s)
Esophageal	3	2	497±108	37±5	579±75	3.8±0.6
Prostate^(c)^	1	2	1316	51	848	5.4
Cervix uteri	1	2	906	49	818	5.0
Rectal	3	1	1259±136	82±8	610±57	4.2±0.3
NPC^(d)^	3	3	668±65	26±1	1418±121	4.5±0.3

a Average leaf travel is calculated over all leaves, excluding leaves which remain closed over all treatment.

b Average leaf travel speed is average leaf travel divided by delivery time.

c The prostate plan is a whole pelvis and prostate boost plan.

d The treatment volume for NPC included all sites: primary, upper, and lower neck with a single VMAT plan.

### B. Measurement devices

Two 3D dosimetry systems, ArcCHECK and ${\text{Delta}}^{4}$, were used for measurements. The ArcCHECK dosimetry system,^(^ ^27^ ^,^ ^31^ ^)^ consists of 1386 diodes, each with $0.8\;\times \;0.8\;{\text{mm}}^{2}$ active measuring area, embedded in the cylindrical wall of the phantom. The ${\text{Delta}}^{4}$ dosimetry system is based on two crossing arrays including 1069 diodes in a fixed cylindrical geometry, providing full coverage of the cross section for any beam direction.^(^ ^28^ ^,^ ^29^ ^)^ The spatial locations of the detectors are different between the two dosimetry systems. The dose distribution tested by ArcCHECK forms a cylindrical distribution with a diameter of 21 cm, typically positioned in the region surrounding the tumor target volume. In the ${\text{Delta}}^{4}$ system, the dose distribution is measured on the two intersected perpendicular planes that cut through the tumor target volume.

### C. Delivery and patient‐specific QA

The 11 VMAT plans were cast on the reference CT images of ArcCHECK and ${\text{Delta}}^{4}$ phantoms and the dose distributions were recalculated. Both 3D diode arrays were placed on the HexaPOD 6D robotic treatment couch (Elekta, Crawley, UK) for measurements. Beam attenuation by the treatment couch has been considered in the plans by generating the couch's model from the contour of the couch, together with the density information. All tests were carried out using an Elekta Synergy accelerator at the nominal energy of 6 MV X‐rays with a 1 cm leaf width MLCi and an RTD 7.01 controller system (Elekta). Before recording QA measurements, we performed the quality control (QC) for the linac according to the TG142 report^(32)^ ensuring the coincidence of lasers with the isocenter was within a radius of 1 mm. To minimize the setup error, cone‐beam computed tomography (CBCT) and a HexaPOD robotic treatment couch (HRTC) were used to set up the phantoms with 0.5 mm and 0.5° residual errors as prescribed in the studies by Sharpe et al.^(33)^ and Meyer et al.^(34)^ The reference CT images were acquired on a CT scanner with a slice thickness of 1 mm, and the resulting CBCTs possessed a voxel resolution of 0.5 mm in all three dimensions of the reconstructed images. Registration between the reference CT and CBCT was carried out automatically using an inbuilt method in XVI, namely gray value match.

### D. Setup error simulation

**Figure 1:**
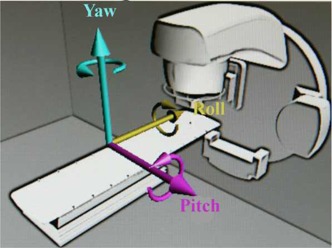
Coordinate system used in the study. Arrows indicate positive rotation with respect to each axis.

The ArcCHECK and ${\text{Delta}}^{4}$ phantoms were translated respectively in the right‐left (RL), anterior‐posterior (AP), and superior‐inferior (SI) directions by ± 1,± 2, and ± 3 mm and rotated in the pitch, roll, and yaw directions by ± 1° and ± 2° using the 6D treatment couch. Figure 1 shows the definition of pitch, roll, and yaw used in this study. The 11 VMAT plans were separately delivered to the each of the two phantoms for dose verification; in total, 31 measurements (1 without positional error, 18 with translational errors, and 12 with rotational errors) were performed for each patient plan with one dosimetry system. For the combined reproducibility of setup and measurement, the above procedure was repeated three times for one rectal cancer case with the ArcCHECK and ${\text{Delta}}^{4}$ systems, respectively. The intervals between the reproducibility tests were more than one month.

We compared the measured dose distributions of each array with the calculated dose distributions generated by the planning system in order to analyze the effect of phantom‐specific setup errors on VMAT QA. We also analyze the cumulative effect of combined 2 mm translational and 1° rotational errors, which could be figured out by the quadratic summation method.^(32)^ The pass rate of γ analysis was computed by comparing the calculated and measured dose distributions using 3%/3 mm, 3%/2 mm, and 2%/2 mm criteria, respectively. Diode readings, or “dose‐values,” lower than 10% of the highest diode signal were ignored in the analysis. These ignored readings reflect low‐dose and low‐gradient regions, typically located under the “jaws,” where the diode response is less reliable and the signal‐to‐noise ratio presents a concern.^(35)^ The paired Student's *t*‐test was used for analysis of the results obtained from ArcCHECK and ${\text{Delta}}^{4}$. All tests were two‐tailed with a p‐value of < 0.05 considered the threshold for statistical significance. Statistical analysis was performed with the SPSS (v.14.0, Chicago, IL) program.

## III. RESULTS

### A. QA for VMAT patients' plans

**Figure 2:**
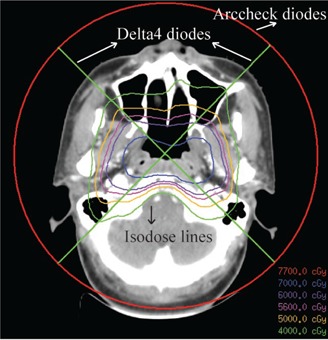
The two systems measured the different section of the dose distribution because of the geometrical position of the diodes. ArcCHECK and ${\text{Delta}}^{4}$ diodes positioned on the circular line and the crossline, respectively.

QA results of the 11 VMAT plans tested with ArcCHECK and ${\text{Delta}}^{4}$ are shown in Table 2. All pass rates of γ analysis with the 3%/3 mm criteria are higher than 95% for both diode arrays. Except for NPC, all pass rates of γ analysis with the 3%/2 mm and 2%/2 mm criteria are higher than 95% and 90%, respectively. The mean pass rate of γ analysis with the 2%/2 mm criteria for NPC by ArcCHECK was 84.7%. The lower results for NPC compared to the other cancer sites are due to the target volume complexity and the differences in the geometrical position of the diodes in ArcCHECK and ${\text{Delta}}^{4}$ (see Fig. 2). A significant difference in the pass rate with the 3%/3mm criteria between the two systems (p = 0.004) indicates that the pass rates of ${\text{Delta}}^{4}$ are higher than those of ArcCHECK.

**Table 2:** The QA results of 11 VMAT plans using ArcCHECK and ${\text{Delta}}^{4}$ phantoms obtained without introducing any setup error

		γ *(%)* ^a^ of ArcCHECK			γ *(%)* ^a^ of ${\text{Delta}}^{4}$
*Cancer Site*	3%/3 mm	3%/2 mm	2%/2 mm	3%/3 mm	3%/2 mm	2%/2 mm
Esophageal	98.5±0.3	96.8±0.4	91.2±0.7	99.7±0.3	98.2±1.0	95.3±1.7
Prostate	98.7	97.5	92.9	98.6	96.5	90.6
Cervix uteri	98.5	97.2	91.4	99.2	98.0	92.9
Rectal	98.8±0.9	97.2±1.8	93.6±2.4	99.4±0.4	97.8±1.2	95.8±1.0
NPC	95.6±0.8	92.5±0.9	84.7±1.0	98.5±0.5	95.3±1.9	90.1±2.0

a Gamma (γ) results are the percentage of points passing the gamma criterion of 3%/3 mm,3%/2 mm, and 2%/2 mm, respectively.

### B. Sensitivities of two diode arrays to translational setup error

**Figure 3:**
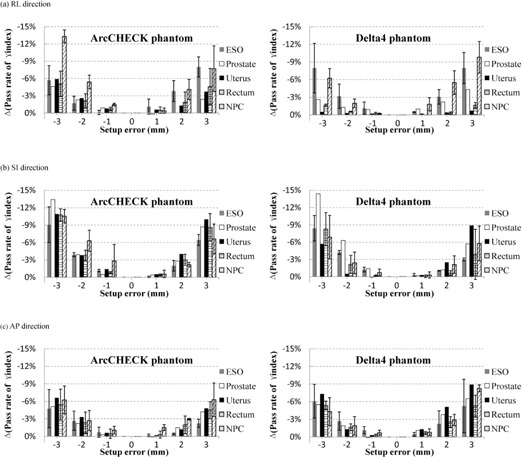
The impact of translational setup errors on dosimetric verification of 11 VMAT plans using ArcCHECK and ${\text{Delta}}^{4}$ phantoms in (a) RL, (b) SI, and (c) AP directions. The simulated translational setup errors are 1, 2, 3, ‐1, ‐2, and ‐3 mm, respectively. The decreased pass rates of γ analysis from the original results are assessed with the 3%/3 mm criteria.

Figure 3 shows the impact of the translational setup error on 11 patient‐specific VMAT QA plans. Setup error was separately introduced in the RL, SI, and AP directions, and the impact was measured using ArcCHECK and ${\text{Delta}}^{4}$. When the translational setup errors are ± 1,± 2, and ± 3 mm, respectively, the pass rates of γ analysis with the 3%/3 mm criteria decreased by a maximum of 2.5%, 6.4%, and 14.0% for ArcCHECK and 2.5%, 6.9%, and 12.2% for ${\text{Delta}}^{4}$ in the RL direction; 6.1%, 8.4%, and 13.4% for ArcCHECK and 1.6%, 6.3%, and 14.4% for ${\text{Delta}}^{4}$ in the SI direction; 2.0%, 4.5%, and 9.5% for ArcCHECK and 1.7%, 5.1%, and 10.5% for ${\text{Delta}}^{4}$ in the AP direction.

To further test the difference between the two dosimetry systems in sensitivity to setup error, we compared all of their values for the reduction of γ analysis with the 3%/3 mm criteria in each direction. Significant differences in the pass rate of γ analysis in the RL and SI directions (p = 0.019 and < 0.001, respectively) indicate a higher sensitivity of ArcCHECK diodes than ${\text{Delta}}^{4}$ diodes to translational setup error in both directions; however, only a nominal difference was observed in the AP direction between the two systems (p = 0.074).

The tested results also indicated that the pass rate of γ analysis was most affected by translation in the RL and AP directions for NPC and esophageal cancer, but only affected by translation in the SI direction for prostate cancer. For ArcCHECK, the maximum standard deviations (error bars shown in Fig. 3) which were calculated for the three cases of each disease site for each setup error were 4.0%, 3.3%, and 3.1%, respectively, for NPC, esophageal, and rectal cancer; for ${\text{Delta}}^{4}$ they were 3.8%, 4.5%, and 4.3%. These results indicate that the effects of translational setup errors on VMAT QA are strongly dependent on patient‐specific plans in spite of the same disease site.

### C. Sensitivities of two diode arrays to rotational setup error

**Figure 4:**
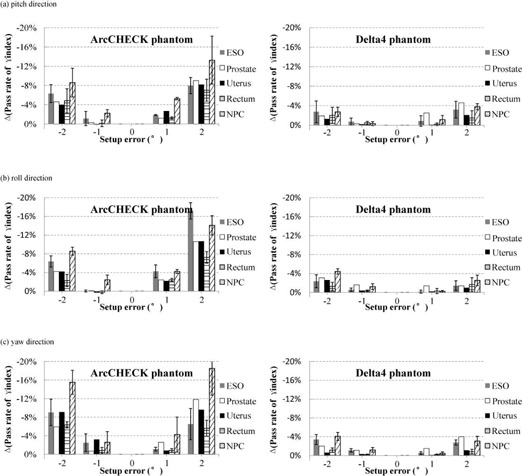
The impact of rotational setup errors on dosimetric verification of 11 VMAT plans using ArcCHECK and ${\text{Delta}}^{4}$ phantoms in (a) pitch, (b) roll, and (c) yaw directions. The simulated rotational setup errors are 1°, 2°, ‐1°, and ‐2°, respectively. The decreased pass rates of γ analysis, compared to the original results, are assessed with the 3%/3 mm criteria.

Figure 4 shows the impact of the rotational setup error for 11 patient‐specific VMAT QA. Setup error was separately introduced in the pitch, roll, and yaw directions, and the impact was measured using ArcCHECK and ${\text{Delta}}^{4}$. When the rotational setup errors were ± 1° and ± 2°, respectively, the pass rates of γ analysis with the 3%/3 mm criteria decreased by a maximum of 5.5% and 9.9% for ArcCHECK and 2.5% and 5.0% for ${\text{Delta}}^{4}$ in the pitch direction; 5.2% and 19.2% for ArcCHECK and 1.8% and 4.9% for ${\text{Delta}}^{4}$ in the roll direction; and 8.4% and 23.5% for ArcCHECK and 1.7% and 4.9% for ${\text{Delta}}^{4}$ in the yaw direction. Significant differences between the two systems in all rotation directions (p < 0.001, = 0.001, and < 0.001 in the pitch, roll, and yaw directions, respectively), indicate that the configuration of the ArcCHECK system is more sensitive to rotational setup error than the ${\text{Delta}}^{4}$ system when determining VMAT QA, and emphasize the importance of accurate rotational positioning during measurements using ArcCHECK.

From the results gathered by the two systems, we observed the greatest impact on the pass rate of γ analysis with the 3%/3 mm criteria in all directions for NPC and esophageal cancer. For ArcCHECK, the maximum standard deviations (error bars shown in Fig. 4) were 5.8%, 3.4%, and 2.5%, respectively, for NPC, esophageal, and rectal cancer; for ${\text{Delta}}^{4}$ they were 1.1%, 2.2%, and 1.6%. These results indicate that the effects of the rotational setup errors on VMAT QA are strongly dependent on patient‐specific plans in spite of the same disease site.

### D. Influence of setup error on the pass rate of γ analysis with various criteria

Table 3 shows the impact of setup errors in translation and rotation on the pass rate of γ analysis with various criteria attained by ArcCHECK and ${\text{Delta}}^{4}$. Stricter gamma criteria resulted in a greater impact of the setup error on the pass rate of γ analysis. For a translational setup error of 3 mm, the pass rates of γ analysis with the 2%/2 mm criteria decreased by an average of 13.2% ± 5.5% for ArcCHECK and by an average of 14.6% ± 6.7% for ${\text{Delta}}^{4}$. For the rotational setup error of 2°, the pass rates of γ analysis with the 2%/2 mm criteria decreased by an average of 14.5% ± 6.6% for ArcCHECK and by an average of 7.0% ± 3.7% for ${\text{Delta}}^{4}$.

**Table 3:** Translational and rotational setup errors result in a decrease in the pass rate of γ analysis with various criteria on the average and standard deviation

		*ArcCHECK*			${\text{Delta}}^{4}$	
*Setup Error*	3%/3 mm	3%/2 mm	2%/2 mm	3%/3 mm	3%/2 mm	2%/2 mm
Translation						
1 mm	0.7±1.0	1.4±1.5	2.0±2.0	0.6±0.6	1.6±1.5	2.1±2.2
2 mm	2.8±1.7	5.0±2.9	6.7±3.5	2.3±1.9	5.4±3.3	7.3±4.2
3 mm	6.9±3.3	10.0±4.9	13.2±5.5	6.1±3.8	11.1±5.5	14.6±6.7
Rotation						
1°	1.8±1.9	3.7±2.9	4.9±3.7	0.5±0.8	1.5±1.5	2.5±2.6
2°	8.6±4.7	12.1±5.5	14.5±6.6	2.4±1.7	5.0±2.9	7.0±3.7

### E. Combined reproducibility of setup and measurement

**Figure 5:**
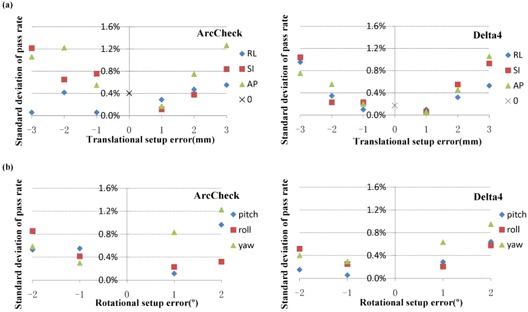
The standard deviation of pass rates with the 3%/3 mm criteria for the reproducibility tests for one rectal cancer case with the ArcCHECK and ${\text{Delta}}^{4}$ systems, respectively. The tests contain all simulated (a) translational and (b) rotational setup errors for this case.

Figure 5 shows the standard deviation of pass rates with the 3%/3 mm criteria for the reproducibility tests for one rectal cancer case with the ArcCHECK and ${\text{Delta}}^{4}$ systems, respectively. The mean standard deviations were 0.59% for ArcCHECK and 0.44% for ${\text{Delta}}^{4}$ for all reproducibility tests in this case, and the mean standard deviations for 1, 2, and 3 mm translational setup errors, respectively, were 0.32%, 0.65%, and 0.83% for ArcCHECK and 0.12%, 0.41%, and 0.88% for ${\text{Delta}}^{4}$; for 1° and 2° rotational setup errors, respectively, they were 0.41% and 0.75% for ArcCheck and 0.29% and 0.54% for ${\text{Delta}}^{4}$.

### F. Cumulative effect of both translational and rotational error

**Figure 6:**
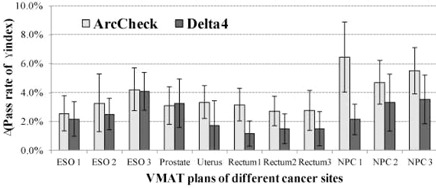
The cumulative impact of 2 mm translational and 1° rotational errors was evaluated for the pass rate reduction with 3%/3 mm criteria for both ArcCHECK and ${\text{Delta}}^{4}$ systems. The calculated average and standard deviation of the pass rate reduction contained the translational and rotational errors of combinations in all directions.

Figure 6 shows the cumulative impact of 2 mm translational and 1° rotational setup errors for the 11 patient‐specific VMAT QA using ArcCHECK and ${\text{Delta}}^{4}$, respectively. For ArcCHECK system, the average decreased pass rates of γ analysis with the 3%/3 mm criteria in all directions were 3.4%, 3.1%, 3.3%, 2.9%, and 5.6%, respectively, for esophageal, prostate, cervix uteri, rectal, and nasopharyngeal cancer, and for ${\text{Delta}}^{4}$ the average decreased pass rates were 2.9%, 3.3%, 1.7%, 1.4%, and 3.0%, respectively.

## IV. DISCUSSION

Patient‐specific dosimetric verification has been indispensable for IMRT QA. Furthermore, achieving accurate QA results is critical in detecting discrepancies between delivery and planning. The characteristics of ${\text{Delta}}^{4}$, as reported by Korreman et al.,^(36)^ were determined by comparing consecutive deliveries of the same plan. The results indicated a strong agreement in all cases for the accumulated dose with dose deviations < 1% for all measurement points and cases. Letourneau et al.^(27)^ assessed the combined reproducibility of the ArcCHECK dosimeter system response and the linear accelerator (Elekta Synergy) for VMAT with the repeat delivery of the head and neck plans. The results demonstrated strong performance and stability of both systems. In our study, we tested the combined reproducibility of setup and measurement. The mean standard deviations were 0.59% (0.06%‐1.27%) for ArcCHECK and 0. 44% (0.06%‐1.06%) for ${\text{Delta}}^{4}$, which shows good reproducibility. However, the results were slightly worse than the reports above because the pass rate altered more significantly as the setup error increased.

Dosimetric verification in the study indicated that the QA of VMAT assessed by both ArcCHECK and ${\text{Delta}}^{4}$ met therapeutic quality requirements. However, it has been noted that the pass rate of γ analysis for ${\text{Delta}}^{4}$ was higher than ArcCHECK, referring to the results for zero setup errors (p = 0.004). The reasons for the differences are as follows. First, ArcCHECK and ${\text{Delta}}^{4}$ dosimetry systems have very different spatial locations of diode detectors. Thus, each system measures a different section of the total dose distribution and samples different dose gradients (see Fig. 2). Compared to ${\text{Delta}}^{4}$, dose distributions of ArcCHECK are more complex. Moreover, ArcCHECK is more sensitive to the dose delivery errors and the angular discretization effect because of the different diode locations.^(37)^ Second, the dose error normalization was different. To establish the percent dose error normalization value, a different method was used for each device, given the different arrangement of the detectors. The ${\text{Delta}}^{4}$ results were normalized at the isocenter, while the ArcCHECK results were normalized to the maximum measured dose in the detector ring. Moreover, the 10% dose cutoff threshold, below which the voxel is excluded from analysis, may not mean the same on the ArcCHECK diode surface as it does on the ${\text{Delta}}^{4}$ diode planes.^(37)^

In all tests of the setup error simulation, the two 3D diode arrays exhibited extreme sensitivity to translational and rotational setup errors in all axes for patient‐specific QA results of VMAT plans, while exhibiting strong dependence on the patient‐specific plan. In general, we have observed an impact of the translational setup error on the QA results of complex VMAT plans and target volumes such as NPC, a cancer contained in the upper and lower neck regions. We have also observed a marked influence of the rotational setup error on the QA results of VMAT plans with long target volumes, such as esophageal cancer. In this paper, we tested three cases for each of the sites of NPC, esophageal, and rectal cancers. Despite the same cancer site amongst case triplets, a difference in sensitivity to setup errors was observed due to the variation of patient‐specific plans. Letourneau et al.^(27)^ assessed the sensitivity of the prototype ArcCHECK dosimetry system for phantom setup error after CBCT image‐guided setup. Letourneau and colleagues found specifically that the diodes' sensitivity to setup error in the RL and AP directions were highly plan‐dependent; the direction of the steepest dose gradients for a given plan did not necessarily correspond with the direction of the phantom setup.

In addition, the results in Figs. 3 and 4 for both sets of setup errors indicate some differences in the order of 3%–4% reduction rates between the negative and positive setup errors. The differences are mainly due to the following two reasons. Firstly, the residual setup errors up to 0.5 mm and 0.5° were still present when CBCT and HRTC were used. Secondly, plan‐specific features along each translational and rotational setup directions were different, such as the dose gradient orientation.

On the other hand, the respective sensitivities to setup error of ArcCHECK and ${\text{Delta}}^{4}$ were not uniform. Though the diode arrays demonstrated similar sensitivity to translational setup error, ArcCHECK was slightly more sensitive than ${\text{Delta}}^{4}$ for the gamma criteria 3%/3 mm, likely measuring a section of the dose distribution with more dose gradients. In addition, compared to the translational setup error, ArcCHECK diodes were more sensitive to the rotational setup error than ${\text{Delta}}^{4}$, due to the difference in spatial locations between the two 3D diode arrays. For the same rotational setup error, the diodes of the ArcCHECK system shift a greater distance than those of ${\text{Delta}}^{4}$.

The AAPM TG‐142 report recommended that the tolerance of laser localization was 1.5 mm for IMRT.^(32)^ For both ArcCHECK and ${\text{Delta}}^{4}$ systems, 1° rotational error could cause an approximate error of 2 mm on the surface of the phantoms. Therefore, the cumulative effect of the combined 2 mm translational and 1° rotational errors was evaluated, and the average pass rates reduction with the 3%/3 mm criteria were 3.8% and 2.4% for ArcCHECK and ${\text{Delta}}^{4}$ systems, respectively. The cumulative effect for ArcCHECK system was more obvious than ${\text{Delta}}^{4}$ system mainly due to the higher sensitivity of ArcCHECK to the rotational error. Especially for NPC tests using ArcCHECK, the cumulative effect was quite obvious; thus, the setup of ArcCHECK should be more accurate than the other cancer sites. In addition, because of the difference in the sensitivity between the two systems, their setup accuracy could be different. As shown in Table 3, in order to achieve less than 3% mean pass rate reduction of VMAT plan QA with the 3%/3 mm criteria, a setup accuracy of 2 mm/1° and 2 mm/2° are required for ArcCheck and ${\text{Delta}}^{4}$ devices, respectively.

## V. CONCLUSIONS

In this study, both the ArcCHECK and ${\text{Delta}}^{4}$ diode arrays showed high sensitivity to setup errors, and sensitivity of both systems is strongly dependent on patient‐specific plans. The sensitivity of ArcCHECK to the rotational error was higher than that of ${\text{Delta}}^{4}$. In order to achieve less than 3% mean pass rate reduction of VMAT plan QA with the 3%/3 mm criteria, a setup accuracy of 2 mm/1° and 2 mm/2° is required for ArcCheck and ${\text{Delta}}^{4}$ devices, respectively. The cumulative effect of the combined 2 mm translational and 1° rotational errors caused 3.8% and 2.4% mean pass rates reduction with 3%/3 mm criteria, respectively, for ArcCHECK and ${\text{Delta}}^{4}$ systems. For QA of VMAT plans for NPC using the ArcCHECK system, the setup should be more accurate.

## ACKNOWLEDGMENTS

Guangjun Li, S en Bai, and Nianyong Chen contributed equally to this work. The ${\text{Delta}}^{4}$ dosimetry system was provided by Beijing HGPT Technology & Trade Co. Ltd. This work was partially supported by the National Natural Science Foundation of China (Grant No. 81101697).

## Supporting information

Supplementary Material
