# Supplementary material for: Evaluation of the sensitivity of two 3D diode array dosimetry systems to setup error for quality assurance (QA) of volumetric‐modulated arc therapy (VMAT)
Source: J Appl Clin Med Phys. 2013 Sep 6;14(5):13–24. doi: 10.1120/jacmp.v14i5.3828 (PMC5714579; doi:10.1120/jacmp.v14i5.3828)
Supplement: Supplementary file 1 — Supplementary Material [file ACM2-14-013-s001.doc]

**Evaluation of the sensitivity of two 3-D diode arrays to setup error for quality assurance (QA) of volumetric modulated arc therapy (VMAT)**

**Abstract:** The purpose of this study is to evaluate the sensitivities of 3-D diode arrays to setup error for patient-specific QA of VMAT. Translation setup errors of ±1, ±2 and ±3 mm in T_X_, T_Y_ and T_Z_ directions and rotation setup errors of ±1° and ±2° in R_X_, R_Y_ and R_Z_ directions were set up in two phantom systems, ArcCHECK and Delta4, with VMAT plans for eleven patients. Cone-Beam CT followed by automatic correction using a Hexapod 6-D treatment couch ensured the position accuracy. Dose distributions of the two phantoms were compared in order to evaluate the agreement between calculated and measured values by using γ analysis with 3%/3 mm, 3%/2 mm, and 2%/2 mm criteria. To determine the impact on setup error for VMAT QA, we evaluated the sensitivity of results acquired by both 3-D diode arrays systems to setup errors in translation and rotation. For all patients’ VMAT QA, the pass rate with 3%/3 mm criteria exceeded 95% using either phantom. For setup errors of 3 mm and 2°, respectively, the pass rates with 3%/3 mm criteria decreased by a maximum of 14.0% and 23.5% using ArcCHECK, and 14.4% and 5.0% using Delta4. The sensitivity of both VMAT QA systems was strongly dependent on the patient-specific plan. In addition, if the standard of eligibility was set within a 3% impact on pass rate using 3%/3 mm criteria, the eligible rates of VMAT QA for all tests with 3 mm translation and 2° rotation setup errors were: for ArcCHECK, 12.1% and 4.5%, respectively; for Delta4, 24.2% and 60.6%, respectively. This demonstrates that both systems are sensitive to setup error, affecting their position accuracy for patient-specific VMAT QA. Therefore, in order to reduce impact on setup error for QA, translation and rotation position errors should be controlled within 2 mm and 1°, respectively.

**Key words:** VMAT, setup error, patient-specific QA, 3-D diode array

**PACS numbers**: 87.55. ne, 87.55.Qr, 87.55. km

**Ⅰ. INTRODUCTION**

Volumetric modulated arc therapy (VMAT) is a new intensity-modulated radiotherapy (IMRT) technology with single or multiple gantry arcs that achieve appropriate dose-target conformity and permit critical organ sparing. VMAT delivers radiation via dynamic multi-leaf collimator (MLC) motion, and allows for variable dose rates, gantry speed modulation and collimator rotation (1). Thus far, VMAT has been used to treat various tumors, including head and neck (2-5), lung (5-8), prostate (4, 5, 9-12), rectum (5, 13), cervix uteri (14), spinal metastases (15, 16), and brain metastases (17). However, the dose calculation and the implementation of VMAT plans are highly complex. It is therefore essential to perform patient-specific quality assurance (QA) of VMAT plans as with IMRT (18).

Various types of dosimetry systems exist for dose verification, including gel dosimetry (19, 20), cylindrical water phantoms with film and ion chambers (21), on-line 2-D detector arrays (22-25), Monte Carlo based framework (26) and 3-D diode arrays (27-29). With the exception of 3-D diode arrays, these QA systems are limited either by single-plane measurements of dose distribution or increased processing time for off-line measurement data (27). Two commercial 3-D diode arrays, ArcCHECK (Sun Nuclear, Melbourne, FL) and Delta4 (ScandiDos AB, Uppsala, Sweden), were applied for dose verification in IMRT and VMAT. ArcCHECK dosimetry system, which has recently been described (27, 30), consists of 1386 diodes, each with 0.8×0.8 mm^2^ active measuring area, embedded in the cylindrical wall of the phantom. Delta4 dosimetry system is based on two crossing arrays including 1069 diodes in a fixed cylindrical geometry, providing full coverage of the cross-section for any beam direction (28, 29). The spatial locations of the detectors are different between the two dosimetry systems. The dose distribution tested by ArcCHECK forms a cylindrical distribution with a diameter of 21 cm, typically positioned in the region surrounding the tumor target volume. In the Delta4, the dose distribution is measured by the intersection of two perpendicular planes that move through the tumor target volume, the region of highest dosage.

In our clinical practice, we used ArcCHECK and Delta4 for QA of VMAT plans. Cross-lines that marked the surface of the phantoms were required to match the laser lines. In practice, we noted that even small coincidence error between the laser crosshairs and the isocenter of linac affected the phantom positioning error, and thereby influenced the dose verification of VMAT. However, unlike in 2-D detector arrays, software for 3-D detector arrays is unable to correct setup error. Therefore, it is crucial to determine the sensitivity of 3-D detector arrays to setup error for VMAT plan QA. In one case, Letourneau et al. (27) assessed the sensitivity of the prototype of the ArcCHECK dosimetry system to phantom translation setup error in RL and AP directions. The resulting data demonstrates that diode array sensitivity to setup error has strong dependence on the patient-specific VMAT plans. However, the effect of rotation setup error on ArcCHECK and other 3-D detector arrays with various detector positions are poorly understood. In this study, we examined the sensitivities of ArcCHECK and Delta4 to translation and rotation setup errors in all directions for patient-specific QA of VMAT plans.

**Ⅱ. METHODS AND MATERIALS**

**A. Patients’ plan selection**

Eleven patients requiring different VMAT planning complexity for cancers including esophageal (ESO), prostate, cervix uteri, rectal and nasopharyngeal cancer (NPC), were selected for this study. VMAT plans were designed using a commercial 3-D treatment planning system (Pinnacle v9.0, Philips Medical, Madison, WI) with a SmartArc optimization algorithm (31). Patients’ characteristics, dose prescription and planning are summarized in Table 1. The planned doses were calculated with a voxel size of 2 mm × 2 mm × 2 mm.

**B. Delivery and patient-specific QA**

The eleven VMAT plans were cast on the reference CT images of ArcCHECK and Delta4 phantoms and the dose distributions were recalculated. Both 3-D diode arrays were placed on the Hexapod 6-D robotic treatment couch (Elekta, Crawley, UK) for measurements. All tests were carried out using an Elekta Synergy accelerator at the nominal energy of 6 MV X-rays with 1cm leaf width MLCi and RTD 7.01 controller system (Elekta, Crawley, UK). Before recording QA measurements, we performed the quality control (QC) for the linac according to TG142 report (32) ensuring the lasers were within a 1 mm sphere of mechanical uncertainty. To minimize setup error, cone-beam CT (CBCT) and hexapod robotic treatment couch (HRTC) were used to setup the phantoms with 0.5 mm and 0.5 degree residual errors as prescribed by references 33 and 34 (33, 34). The reference CT images were acquired on a CT scanner with a slice thickness of 1mm, and the resulting CBCTs possessed a voxel resolution of 0.5 mm in all three dimensions of the reconstructed images. Registration between reference CT and CBCT was carried out automatically using an inbuilt method in XVI, namely gray value match.

**C. Setup error simulation**

The ArcCHECK and Delta4 phantoms were translated respectively in T_X_ (translation along X axis, i.e. perpendicular to the axes of both gantry and couch rotation), T_Y_ (translation along Y axis, i.e. parallel to the axis of gantry rotation) and T_Z_ (translation along Z axis, i.e. parallel to the axis of couch rotation) directions by ±1, ±2 and ±3 mm and rotated in R_X_, R_Y_ and R_Z_ (single-axis rotations around X, Y and Z axes) directions by ±1° and ±2° using the 6-D treatment couch (see Fig. 1). The eleven VMAT plans were separately delivered to the each of the two phantoms for dose verification; in total, 31 measurements were performed for each of the eleven plans and each of the two systems.

We compared the measured dose distributions of each array with the calculated dose distributions generated by the planning system in order to analyze the effect of phantom-specific setup error on VMAT QA. The pass rate of γ analysis was computed by comparing calculated and measured dose distributions using 3%/3 mm, 3%/2 mm and 2%/2 mm criteria, respectively. Diode readings, or “dose-values,” lower than 10*%* of the highest diode signal were ignored in the analysis. These ignored readings reflect low-dose and low-gradient regions, typically located under the “jaws,” where diode response is less reliable and signal to noise ratio presents concern (35). The paired Student t-test was used for analysis of results obtained from ArcCHECK and Delta4. All tests were two-tailed with a *p* value of <0.05 considered the threshold for statistical significance. Statistical analysis was performed with SPSS (v.14.0, Chicago, IL) program.

**Ⅲ. RESULTS**

**A. QA for VMAT patients’ plans**

QA results of the eleven VMAT plans tested with ArcCHECK and Delta4 are shown in Table 2. All pass rates of γ analysis with 3%/3 mm criteria are higher than 95% for both diode arrays. Except NPC, all pass rates of γ analysis with 3%/2 mm and 2%/2 mm criteria are higher than 95% and 90%, respectively. The mean pass rate of γ analysis with 2%/2 mm criteria for NPC by ArcCHECK was 84.7%. The lower results for NPC compared to other cancer sites are due to the target volume complexity and the differences in geometrical position of the diodes in ArcCHECK and Delta4 (see Fig. 2).

**B. Sensitivities of two diode arrays to translation setup error**

Figure 3 shows the impact of translation setup error upon eleven patient-specific VMAT QA plans. Setup error was separately introduced in T_X_, T_Y_ and T_Z_ directions, and the impact was measured using ArcCHECK and Delta4. When the translation setup errors are ±1, ±2 and ±3 mm, respectively, the pass rates of γ analysis with 3%/3 mm criteria decreased by a maximum of: 2.5%, 6.4% and 14.0% for ArcCHECK and 2.5%, 6.9% and 12.2% for Delta4 in T_X_ direction; 6.1%, 8.4% and 13.4% for ArcCHECK and 1.6%, 6.3% and 14.4% for Delta4 in T_Y_ direction; 2.0%, 4.5% and 9.5% for ArcCHECK and 1.7%, 5.1% and 10.5% for Delta4 in T_Z_ direction.

To further test the difference between the two dosimetry systems in sensitivity to setup error, we compared all of their values for the reduction of γ analysis with 3%/3mm criteria in each direction. Significant differences in the pass rate of γ analysis in T_X_ and T_Y_ directions (p values 0.019 and 0.000, respectively) indicate a higher sensitivity of ArcCHECK diodes than Delta4 diodes to translation setup error in both directions; however, only nominal difference was observed in T_Z_ direction between the two systems (p value 0.074).

The tested results also indicated that the pass rate of γ analysis was most affected by translation in T_X_ and T_Z_ directions for NPC and ESO, but only affected by translation in T_Y_ direction for prostate cancer. For ArcCHECK, the maximum standard deviations of translation setup error in all directions for NPC, ESO and rectal cancer were 4.0%, 3.3% and 3.1%, respectively; for Delta4 they were 3.8%, 4.5% and 4.3%.

**C. Sensitivities of two diode arrays to rotation setup error**

Figure 4 shows the impact of rotation setup error for eleven patient-specific VMAT QA. Setup error was separately introduced in R_X_, R_Y_ and R_Z_ directions, and the impact was measured using ArcCHECK and Delta4. When the rotation setup errors were ±1° and ±2°, respectively, the pass rates of γ analysis with 3%/3 mm criteria decreased by a maximum of: 5.5% and 9.9% for ArcCHECK and 2.5% and 5.0% for Delta4 in R_X_ direction; 5.2% and 19.2% for ArcCHECK and 1.8% and 4.9% for Delta4 in R_Y_ direction; 8.4 and 23.5% for ArcCHECK and 1.7% and 4.9% for Delta4 in R_Z_ direction. Significant differences between the two systems in all rotation directions (p values 0.000, 0.001 and 0.000 in R_X_, R_Y_ and R_Z_ directions, respectively), indicates that ArcCHECK diodes are more sensitive to rotation setup error than Delta4 diodes when determining VMAT QA, emphasizing the importance of QA during measurements using ArcCHECK.

From the results gathered by the two systems, we observed the greatest impact on pass rate of γ analysis with 3%/3 mm criteria in all directions for NPC and ESO. For ArcCHECK, the maximum standard deviations of rotation setup error in all directions for NPC, ESO and rectal cancer were 5.8%, 3.4% and 2.5%, respectively; for Delta4 they were 1.1%, 2.2% and 1.6%. These results suggest that the effects of rotational setup errors on VMAT QA are strongly dependent on patient-specific plans.

**D. Influence of setup error on the pass rate of γ analysis with various criteria**

Table 3 shows the impact of setup errors in translation and rotation on the pass rate of γ analysis with various criteria attained by ArcCHECK and Delta4. Stricter gamma criteria resulted in greater impact of setup error on the pass rate of γ analysis. For translation setup error of 3 mm, the pass rates of γ analysis with 2%/2 mm criteria decreased by an average of 13.2±5.5% for ArcCHECK and by an average of 14.6±6.7% for Delta4. For rotation setup error of 2°, the pass rates of γ analysis with 2%/2 mm criteria decreased by an average of 14.5±6.6% for ArcCHECK and by an average of 7.0±3.7% for Delta4.

We assume a 3% eligibility standard for impact of setup error on the pass rate of γ analysis with 3%/3 mm criteria. The qualification rates of VMAT QA for all tests with a setup error of 1, 2 and 3 mm in translation and of 1° and 2° in rotation were as follows: for ArcCHECK, 97.0%, 60.6% and 12.1% (translation) and 78.8% and 4.5% (rotation), respectively; for Delta4, 100%, 69.7% and 24.2% (translation) and 100% and 60.6% (rotation), respectively. These results indicate that more stringent criteria result in lower γ analysis pass rates (as shown in Table 3).

**Ⅳ. DISCUSSION**

Patient-specific dosimetric verification has been indispensable for IMRT QA. Furthermore, achieving accurate QA results is critical in detecting discrepancies between delivery and planning. The characteristics of Delta4 have been reported by Korreman et al. (36), determined by comparing consecutive deliveries of the same plan. The tested result indicated a strong agreement in all cases for the accumulated dose with dose deviations < 1% for all measurement points and cases. Letourneau et al. (27) assessed the combined reproducibility of ArcCHECK dosimeter system response and the linear accelerator (Elekta Synergy) for VMAT with the repeat delivery of the head and neck plans. The results demonstrated strong performance and stability of both systems.

Dosimetric verification in the study indicated that the QA of VMAT assessed by both ArcCHECK and Delta4 met therapeutic quality requirements. However, it has been noted that the pass rate of γ analysis for Delta4 was typically found to be higher than ArcCHECK (as shown in Table 2). The reasons for the differences are as follows. First, ArcCHECK and Delta4 dosimetry systems have very different spatial locations of diode detectors. Thus each system measures a different section of the total dose distribution and samples different dose gradients (see Fig. 2). Delta4 mainly samples the dose distribution in the target volume, while ArcCHECK samples outside the target volume. Compared to Delta4, dose distributions of ArcCHECK are more complex, and high dose gradient regions are more extensive. Second, the calculated dose away from isocenter is unstable with respect to the angular discretization effects, while the central region’s dose is more stable. Because of the different diode locations, the ArcCHECK is more sensitive to the angular discretization effect (37). Finally, the dose error normalization cannot be the same. The 10% dose cut-off threshold, below which the voxel is excluded from analysis, may have different implications on the ArcCHECK diode surface than on the Delta4 diode planes (37).

In all tests of setup error simulation, the two 3-D diode arrays exhibited extreme sensitivity to translation and rotation setup errors in all axes for patient-specific QA results of VMAT plans, while exhibiting strong dependence on the patient-specific plan. In general, we have observed an impact of translation setup error on the QA results of complex VMAT plans and target volumes such as NPC, a cancer contained in the upper and lower neck regions. We have also observed a marked influence of rotation setup error on the QA results of VMAT plans with long target volumes such as ESO. In this paper, we tested three cases for each of the sites of NPC, ESO and rectal cancers. Despite the same cancer site amongst case triplets, a difference in sensitivity to setup errors was observed due to the variation of patient-specific plans. Letourneau et al. (27) assessed the sensitivity of the prototype ArcCHECK dosimetry system for phantom setup error after CBCT image-guided setup. Letourneau found specifically that the diodes’ sensitivity to setup error in RL and AP directions were highly plan-dependent; the direction of steepest dose gradients for a given plan did not necessarily correspond with the direction of the phantom setup.

On the other hand, the respective sensitivities to setup error of ArcCHECK and Delta4 were not uniform. Though the diode arrays demonstrated similar sensitivity to translation setup error, ArcCHECK was slightly more sensitive than Delta4 for gamma criteria 3%/3 mm, likely measuring a section of the dose distribution with more dose gradients. In addition, compared to translation setup error, ArcCHECK diodes were more sensitive to rotation setup error than Delta4, due to the difference in spatial locations between the two 3-D diode arrays and, likely, the gantry angle as well. For the same rotation setup error, the diodes of ArcCHECK system shift a greater distance than those of Delta4.

In addition, our data indicate an extreme sensitivity of diode arrays to setup error as shown in Table 3. Slight increase in setup error resulted in a sharp drop in qualification rates for VMAT QA. Thus, before performing patient-specific QA of VMAT plans, it must be ensured that translation and rotation setup errors are within 2 mm and 1°, respectively.

**Ⅴ. CONCLUSION**

In this study, both the ArcCHECK and Delta4 diode arrays showed high sensitivity to setup errors. As a result, translation and rotation setup errors should be within 2 mm and 1°, respectively, to reduce their impact upon patient-specific QA of VMAT plans. Thus, in order to ensure the accuracy of phantom setup before VMAT QA, periodic linac QA/QC should first be performed and the laser position and angle subsequently verified.

**ACKNOWLEDGEMENTS**

Delta4 dosimetry system was provided by Beijing HGPT Technology & Trade Co. Ltd. This work was partially supported by National Natural Science Foundation of China (Grant No. 81101697).

**REFERENCES**

1. Otto K. Volumetric modulated arc therapy: IMRT in a single arc. Med Phys. 2008; 35: 310–317.

2. Verbakel WF, Cuijpers JP, Hoffmans D, Bieker M, Slotman BJ, Senan S. Volumetric intensity-modulated arc therapy vs. conventional IMRT in head-and-neck cancer: a comparative planning and dosimetric study. Int J Radiat Oncol Biol Phys. 2009; 74**:** 252–259.

3. Bertelsen A, Hansen CR, Johansen J, Brink C. Single Arc Volumetric Modulated Arc Therapy of head and neck cancer. Radiother Oncol. 2010; 95: 142-148.

4. Yu C, Li X, Ma L, Chen D, Naqvi S, Shepard D, Sarfaraz M, Holmes T, Suntharalingam M, Mansfield C. Clinical implementation of intensity-modulated arc therapy. Int J Radiat Oncol Biol Phys. 2002; 53**:** 453–463.

5. Cao D, Holmes T, Afghan M, Shepard D. Comparison of plan quality provided by intensity-modulated arc therapy and helical tomotherapy. Int J Radiat Oncol Biol Phys. 2007; 69: 240–250.

6. Bedford J, Hansen V, McNair H, Aitken A, Brock J, Warrington A, Brada M. Treatment of lung cancer using volumetric modulated arc therapy and image guidance: a case study. Acta Oncol. 2008; 47: 1438–1443.

7. Verbakel WF, Senan S, Cuijpers JP, Slotman BJ, Lagerwaard FJ. Rapid delivery of stereotactic radiotherapy for peripheral lung tumors using volumetric intensity-modulated arcs. Radiother Oncol. 2009; 93: 122-124.

8. McGrath SD, Matuszak MM, Yan D, Kestin LL, Martinez AA, Grills IS. Volumetric modulated arc therapy for delivery of hypofractionated stereotactic lung radiotherapy: A dosimetric and treatment efficiency analysis. Radiother Oncol. 2010; 95: 153-157.

9. Ma L, Yu C, Earl M, Holmes T, Sarfaraz M, Li X, Shepard D, Amin P, DiBiase S, Suntharalingam M, Mansfield C. Optimized intensity-modulated arc therapy for prostate cancer treatment. Int J Cancer. 2001; 96:379–384.

10. Palma D, Vollans E, James K, Nakano S, Moiseenko V, Shaffer R, Mckenzie M, Morris J, Otto K. Volumetric modulated arc therapy for delivery of prostate radiotherapy: comparison with intensity-modulated radiotherapy and three-dimensional conformal radiotherapy. Int J Radiat Oncol Biol Phys. 2008; 72: 996–1001.

11. Wolff D, Stieler F, Welzel G, Lorenz F, Madyan Y, Mai S, Herskind C, Polednik M, Steil V, Wenz F, Lohr F. Volumetric modulated arc therapy (VMAT) vs. serial tomotherapy, step-and-shoot IMRT and 3-D-conformal RT for treatment of prostate cancer. Radiother Oncol. 2009; 93: 226-233.

12. Guckenberger M, Richter A, Krieger T, Wilbert J, Baier K, Flentje M. Is a single arc sufficient in volumetric-modulated arc therapy (VMAT) for complex-shaped target volumes? Radiother Oncol. 2009; 93: 259-265.

13. Duthoy W, Gersem W, Vergote K, Boterberg T, Derie C, Smeets P, Wagter C, Neve W. Clinical implementation of intensity-modulated arc therapy (IMAT) for rectal cancer. Int J Radiat Oncol Biol Phys. 2004; 60: 794–806.

14. Cozzi L, Dinshaw KA, Shrivastava SK, Mahantshetty U, Engineer R, Deshpande DD, Jamema SV, Vanetti E, Clivio A, Nicolini G, Fogliata A. A treatment planning study comparing volumetric arc modulation with RapidArc and fixed field IMRT for cervix uteri radiotherapy. Radiother Oncol. 2008; 89: 180-191.

15. Matuszak M, Sui H, Yan D. Potential impact of volumetric modulated arc therapy on the planning and delivery of radiation therapy. Int J Radiat Oncol Biol Phys. 2008; 72: S651.

16. Kuijper IT, Dahele M, Senan S, Verbakel W. Volumetric modulated arc therapy versus conventional intensity modulated radiation therapy for stereotactic spine radiotherapy: A planning study and early clinical data. Radiother Oncol. 2010; 94: 224-228.

17. Clark GM, Popple RA, Young PE, Fiveash JB. Feasibility of single-isocenter volumetric modulated arc radiosurgery for treatment of multiple brain metastases. Int J Radiat Oncol Biol Phys. 2010; 76: 296-302.

18. Bortfeld T, Webb S. Single-arc IMRT? Phys Med Biol. 2009; 54: N9–N20.

19. Low D, Dempsey J, Venkatesan R, Mutic S, Markman J, Haacke E, Purdy J. Evaluation of polymer gels and MRI as a 3-D dosimeter for intensity-modulated radiation therapy. Med Phys. 1999; 26: 1542–1551.

20. Vergote K, Deene YD, Duthoy W, Gersem WD, Neve WD, Achten E, Wagter CD. Validation and application of polymer gel dosimetry for the dose verification of an intensity-modulated arc therapy (IMAT) treatment. Phys Med Biol. 2004; 49: 287–305.

21. Pallotta S, Marrazzo L, Bucciolini M. Design and implementation of a water phantom for IMRT, arc therapy, and tomotherapy dose distribution measurements. Med Phys. 2007; 34: 3724–3731.

22. Jursinic P, Nelms B. A 2-D diode array and analysis software for verification of intensity modulated radiation therapy delivery. Med Phys. 2003; 30: 870–879.

23. Spezi E, Angelini A, Romani F, Ferri A. Characterization of a 2D ion chamber array for the verification of radiotherapy treatments. Phys Med Biol. 2005; 50: 3361–3373.

24. Poppe B, Blechschmidt A, Djouguela A, Kollhoff R, Rubach A, Willborn K, Harder D. Two-dimensional ionization chamber arrays for IMRT plan verification. Med Phys. 2006; 33: 1005–1015.

25. Jursinic PA, Sharma R, Reuter J. MapCHECK used for rotational IMRT measurements: Step-and-shoot, Tomotherapy, RapidArc. Med Phys. 2010; 37: 2837-2846.

26. Bush K, Townson R, Zavgorodni S. Monte Carlo simulation of RapidArc radiotherapy delivery. Phys Med Biol. 2008; 53: N359–N370.

27. Letourneau D, Publicover J, Kozelka J, Moseley DJ, Jaffray DA. Novel dosimetric phantom for quality assurance of volumetric modulated arc therapy. Med Phys. 2009; 36: 1813-1821.

28. Bedford J, Lee Y, Wai P, South C, Warrington A. Evaluation of the Delta4 phantom for IMRT and VMAT verification. Phys Med Biol. 2009; 54: N167–N176.

29. Sadagopan R, Bencomo J, Martin R, Nilsson G, Matzen T, Balter P. Characterization and clinical evaluation of a novel IMRT quality assurance system. J Appl Clin Med Phys. 2009; 10: 104–119.

30. Yan G, Lu B, Kozelka J, Liu C, Li JG. Calibration of a novel four-dimensional diode array. Med Phys. 2010; 37: 108-115.

31. Bzdusek K, Friberger H, Eriksson K, Hardemark B, Robinson D, Kaus M. Development and evaluation of an efficient approach to volumetric arc therapy planning. Med Phys. 2009; 36: 2328–839.

32. Klein EE, Hanley J, Bayouth J, Yin F, Simon W, Dresser S, Serago C, Aguirre F, Ma L, Arjomandy B, Liu C. Task Group 142 Report: Quality Assurance of Medical Accelerators. Med Phys. 2009; 36: 4197–4212.

33. Sharpe MB, Moseley DJ, Purdie TG, Islam M, Siewerdsen J, Jaffray D. The stability of mechanical calibration for a kV cone beam computed tomography system integrated with linear accelerator. Med Phys. 2006; 33: 136–144.

34. Meyer J, Wilbert J, Baier K, Guckenberger M, Richter A, Sauer O, Flentje M. Positioning accuracy of cone-beam computed tomography in combination with a hexapod robot treatment table. Int J Radiat Oncol Biol Phys. 2007; 67: 1220-1228.

35. Basran PS, Woo MK. An analysis of tolerance levels in IMRT quality assurance procedures. Med Phys. 2008; 35: 2300-2307.

36. Korreman S, Medin J, Kristoffersen FK. Dosimetric verification of RapidArc treatment delivery. Acta Oncol. 2009; 48: 185-191.

37. Feygelman V, Zhang G, Stevens C, Nelms BE. Evaluation of a new VMAT QA device, or the “X” and “O” array geometries. J Appl Clin Med Phys. 2011; 12: 146–168.

**Figure Legends**

FIG. 1. Coordinate system indicating both the three translational axes and the rotational axes.

FIG. 2. The two systems measured the different section of the dose distribution because of the geometrical position of the diodes, and sampled the different dose gradients on transverse view for one representative NPC case. (a) Arccheck and Delta4 diodes positioned on the circular line and the cross-line, respectively; (b) and (c) illustrate the measured and calculated dose distributions on the slice for Arccheck and Delta4, respectively.

**FIG. 3.** The impact of translation setup errors upon dosimetric verification of eleven VMAT plans using ArcCHECK and Delta4 phantom in (a) T_X_, (b) T_Y_ and (c) T_Z_ directions. The simulated translation setup errors are 1, 2, 3, -1, -2 and -3 mm, respectively. And the decreased pass rates of γ analysis from original results are assessed with 3%/3 mm criteria.

**FIG. 4.** The impact of rotation setup errors upon dosimetric verification of eleven VMAT plans using ArcCHECK and Delta4 phantom in (a) R_X_, (b) R_Y_ and (c) R_Z_ directions. The simulated rotation setup errors are 1°, 2°, -1° and -2°, respectively. The decreased pass rates of γ analysis, compared to original results, are assessed with 3%/3 mm criteria.

**Table Captions**

**TABLE. 1.** Patient characteristics, dose prescription and planning states. Using the SIB technique, two or three target volumes are defined for each patient except for rectal cancer.

**TABLE. 2.** The QA results of eleven VMAT plans using ArcCHECK and Delta4 phantom obtained without introducing any setup error.

**TABLE. 3.** Translation and rotation setup errors make an impact on the pass rate of γ analysis with various criteria, which is attained by ArcCHECK and Delta4 phantom.
